# The human renal lymphatics under normal and pathological conditions

**DOI:** 10.1111/j.1365-2559.2006.02478.x

**Published:** 2005-09-12

**Authors:** Y Ishikawa, Y Akasaka, H Kiguchi, Y Akishima-Fukasawa, T Hasegawa, K Ito, M Kimura-Matsumoto, S Ishiguro, H Morita, S Sato, S Soh, T Ishii

**Affiliations:** Department of Pathology, Toho University School of Medicine Tokyo; 1 Department of Pathology, Saiseikai Kanagawaken Hospital Yokohama; 2 Pathology Division, National Cancer Centre Research Institute, Toho University Medical Centre Ohmori Hospital Tokyo; 3 Department of Gastroenterology and Hepatology, Toho University Medical Centre Ohmori Hospital Tokyo; 4 Department of Urology, Kitasato University School of Medicine Kanagawa, Japan

**Keywords:** D2-40, immunohistochemistry, renal anatomy, renal cell carcinoma, renal lymphatics

## Abstract

Ishikawa Y, Akasaka Y, Kiguchi H, Akishima-Fukasawa Y, Hasegawa T, Ito K, Kimura-Matsumoto M, Ishiguro S, Morita H, Sato S, Soh S & Ishii T (2006) *Histopathology* **49**, 265–273

**The human renal lymphatics under normal and pathological conditions**

**Aims:**

The renal lymphatics have not been fully documented in humans. The aim of this study was to clarify the morphology of the human renal lymphatic system under normal and pathological conditions by immunohistochemistry using anti-D2-40 antibody.

**Methods and results:**

Normal and pathological renal tissues obtained at autopsy as well as nephrectomy specimens with renal cell carcinoma (RCC) were used. Thin sections were immunostained with antibodies against D2-40 and CD31. In normal kidney, D2-40+ lymphatics were abundant in the interstitium around the interlobar and arcuate arteries/veins but sporadic in those around the glomeruli or between the tubules in the cortex. A few lymphatics contained erythrocytes in their lumina. Lymphatics were seldom present in the medulla. In RCC cases, lymphatics were evident at the tumour margin, whereas CD31+ capillaries were abundant throughout the tumour and lymphatics were increased in the fibrous interstitium around the tumour. Lymphatic invasion by RCC cells was also detectable. D2-40+ lymphatics were evident in other pathological conditions and end-stage kidney had a denser lymphatic distribution than normal kidney.

**Conclusions:**

Lymphatics are abundant around the arteries/veins and are also present in the renal cortex and medulla. D2-40 immunostaining is helpful for investigating the pathophysiological role of renal lymphatics.

## Introduction

The renal lymphatics contribute to the draining of excess fluid and protein under both normal and abnormal physiological conditions.^1^ The distribution of the normal renal lymphatic system has been investigated in various animals using light microscopy by the ureteric occlusion technique,^2^ electron microscopy^3^,^4^ and microradiography.^4^ In humans, microradiography has been performed to detect the renal lymphatics at autopsy.^5^ So far, however, there has been no comprehensive light-microscopic study of the lymphatic system in the normal human kidney, due to the lack of a reliable marker that can distinguish the lymphatic endothelium from the blood capillary endothelium. A previous report has described the lymphatic distribution in the human kidney, where lymphatics were clearly visible due to their distension caused by widespread permeation of gastric cancer in their lumina.^6^

In recent years, certain lymphatic endothelial cell-specific proteins have been identified, including vascular endothelial growth factor receptor-3, lymphatic endothelial hyaluronan receptor-1 (LYVE-1), podoplanin, prox-1, D6 and D2-40.^7^,^8^ Among these, an antibody to D2-40 has been shown to detect lymphatic endothelium in formalin-fixed paraffin-embedded tissues and does not react with the blood vessel endothelium.^8^ This antibody has already been employed to detect lymphatics in various tissues,^9^,^10^ but there has been no report on the lymphatic distribution of the human kidney using such lymphatic markers, except for a recent study using biopsy specimens from transplanted kidneys.^11^

In light of the above issues, we carried out immunohistochemical studies with anti-D2-40 to elucidate the lymphatics in relation to the overall anatomical structure of the normal human kidney and in various pathological conditions, including renal cell carcinoma (RCC), end-stage kidney from haemodialysis patients, cortical infarction, acute tubular necrosis and hydronephrosis.

## Materials and methods

Normal renal tissues were obtained from 10 autopsy cases within 3 h of death, the average patient's age being 67.5 ± 8.2 years (five men and five women). For the normal cases, two pieces of tissue per case were dissected (total, 20 sections). These samples were confirmed microscopically to contain normal structures, with the exception of mild arteriosclerosis or congestion. Ten specimens obtained by nephrectomy for RCC were also collected from patients aged 61.8 ± 10.6 years (seven men and three women). All the RCCs exhibited clear cell carcinoma with a fibrous capsule and 40 samples of renal tissue including tumour-free regions were obtained from these specimens. Three cases of end-stage kidney from haemodialysis patients (aged 59.9 ± 8.2 years; two men and one woman), two cases of cortical infarction (from men aged 68 and 75 years), one case of acute tubular necrosis (from a 58-year-old man) and two cases of hydronephrosis (from men aged 56 and 66 years) were also examined. These eight specimens of diseased kidney were obtained at autopsy within 3 h of death; two pieces of renal tissue per case were dissected out.

A total of 76 renal tissue samples were fixed with 10% neutral-buffered formaldehyde and embedded in paraffin. Thin-sections were then cut, and stained with haematoxylin and eosin, Azan–Mallory and elastica van Gieson. Subserial sections were immunostained with anti-D2-40 antibody (Signet Laboratory Inc., Dedham, MA, USA) using a CSA II kit (DakoCytomation Inc., Carpinteria, CA, USA) according to the manufacturer's instructions. Sections obtained from normal kidneys and kidneys affected by RCC were additionally immunostained with anti-CD31 antibody (DakoCytomation Inc.) by the same method as that used for D2-40. Sections were visualized by treating the slides with diaminobenzidine tetrahydrochloride. To verify antibody specificity, sections from each paraffin block were used as negative controls by omitting the primary antibody and replacing it with normal mouse immunoglobulin.

In addition, for the confirmation of D2-40 specificity for lymphatic endothelia, normal gastric sections obtained from two autopsy cases were immunostained with antibodies for D2-40 and von Willebrand factor (DakoCytomation Inc.) using the CSA II kit (DakoCytomation Inc.).

The distribution of lymphatics in normal kidneys was analysed statistically in 20 sections from the following five categorized anatomical locations: the cortical interstitium between the tubules, the interstitium around the glomeruli, the medullary interstitium between the tubules and/or collecting ducts, the interstitium around the interlobular arteries/veins in the cortex, and the interstitium around the interlobar arteries/veins and the arcuate arteries/veins, respectively. The sections immunostained for D2-40 were observed at random by light microscopy at high magnification (× 200). The number of D2-40+ lymphatics was counted in each × 200 field and the total number of lymphatics in each field of view was used for statistical analysis.

The number of lymphatics in RCC cases and end-stage kidneys was also analysed statistically. In RCC cases, the number of lymphatics in the tumour as well as in the fibrous cortex around it was counted in the same way as for normal cases. In cases of end-stage kidney, the number of lymphatics in the cortex was counted using the same methods. In these pathological cases, the number of lymphatics in the cortex, except for the interstitium around the interlobar and arcuate vessels, was counted. The extent of lymphatic distribution in such cases was compared statistically with that at the same location in the normal kidney.

## Results

### D2-40 specificity for lymphatics

**Figure 1:**
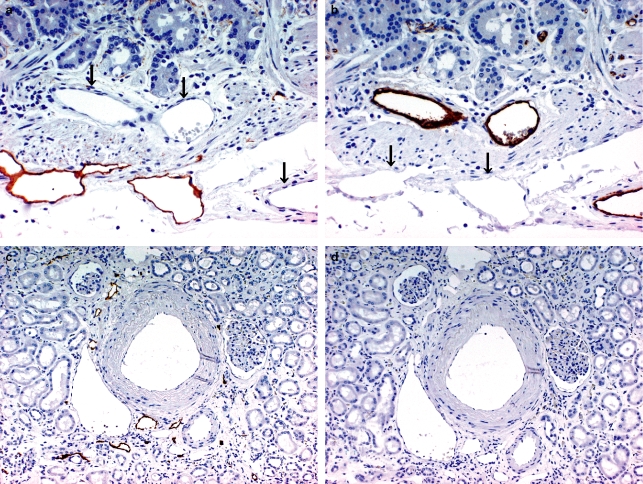
D2-40 and von Willebrand factor immunostaining of gastric and renal tissues. **a**, D2-40 immunostaining of gastric tissue. D2-40 is positive in lymphatic capillaries just under the muscularis mucosae, but negative in blood vessels indicated by arrows. **b**, Immunohistochemistry with von Willebrand factor in a serial section of that seen in (**a**). Blood vessels indicated by arrows in (**a**) are positive, but lymphatic capillaries indicated by arrows in this figure are negative. **c**, D2-40 immunostaining of the kidney. Capillaries around the arteries in the cortex are positive for D2-40 antibody, but the endothelia of the arteries and glomeruli are negative. **d**, Negative control section of (**c**) by omitting the primary antibody and replacing it with normal mouse immunoglobulin. The figure is a serial section of that seen in (**c**), which demonstrates negative staining.

In general, lymphatics are abundant in the interstitium around the muscularis mucosae of normal stomach, which are positive for lymphatic markers such as LYVE-1.^7^ D2-40 was detected on the endothelia of lymphatics around the musclaris mucosae of gastric tissue but not in blood vessels (Figure 1a). In contrast, those lymphatics positive for D2-40 were negative for von Willebrand factor, which was found in the endothelial cells of blood vessels (Figure 1b). In normal kidney, the endothelial cells of blood vessels and glomeruli were negative for D2-40 (Figure 1c). Renal sections used as negative controls by omitting D2-40 antibody and replacing it with normal mouse immunoglobulin were not stained (Figure 1d).

### Lymphatic distribution in the normal kidney

#### Renal cortex

**Figure 2:**
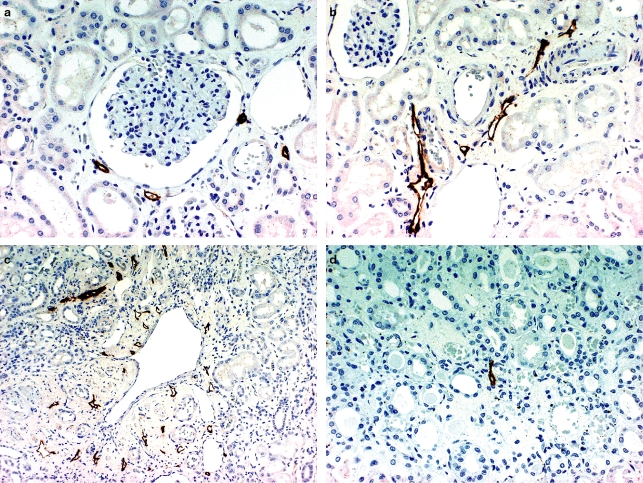
Lymphatics in the normal kidney (D2-40 immunostaining). **b**, Lymphatic capillaries are evident in the interstitium around the glomerulus. **c**, Lymphatics exhibiting a slit-like structure are distributed around the interlobular artery and vein in the cortex. **c**, In the interstitium around a dilated interlobular vein, many lymphatic capillaries are evident. A few lymphatic capillaries are present just beneath the venous endothelium. **d**, A lymphatic capillary is recognizable in the centre of the figure showing a normal medulla.

In the interstitium between the tubules, lymphatic capillaries exhibited a tubular shape or a slit-like structure and were scarce near blood capillaries. Similar lymphatic capillaries were observed sporadically in the interstitium around the glomeruli (Figure 2a). The lumina of lymphatic capillaries between the tubules or around the glomeruli were empty and they were fewer in number than blood capillaries. Lymphatic capillaries positive for D2-40 were sometimes also positive for CD31. The interlobular arteries and veins were accompanied by a relatively narrow and fibrous interstitium, in which lymphatic vessels were abundant (Figure 2b). Lymphatics around the interlobular veins were more developed than those around the interlobular arteries (Figure 2c). Blood vessel-related lymphatics observed in the cortex generally had empty lumina but sometimes contained a few lymphocytes or erythrocytes.

#### Renal medulla

Lymphatics were extremely rare in the medulla. Among the 10 normal cases, lymphatic capillaries were found in only four (Figure 2d) and occurred mainly in the interstitium between the ducts near the cortex, but not in the central area of the medulla.

#### Large blood vessel-related lymphatics

**Figure 3:**
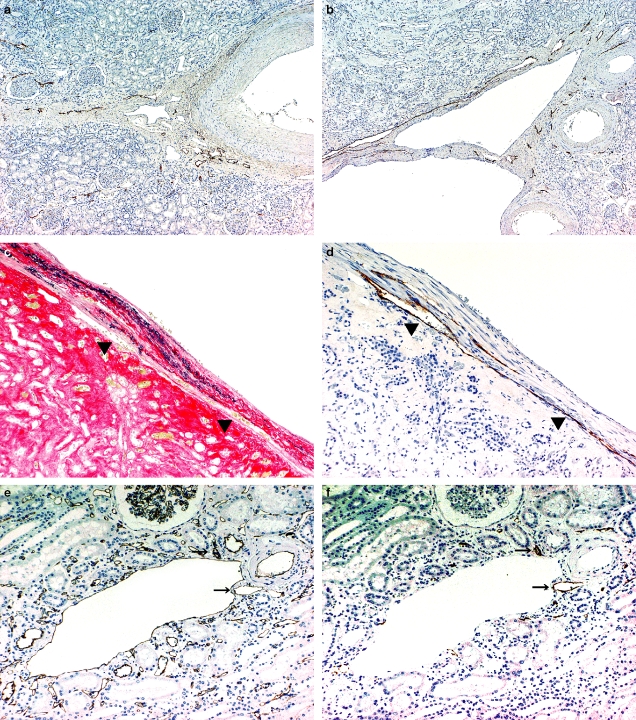
Large blood vessel-related lymphatics in the normal kidney. **a**, Lymphatic vessels positive for D2-40 are abundantly distributed in the interstitium around the interlobar artery. **b**, Many lymphatics are shown around the interlobar veins. Longitudinal aspect of a lymphatic vessel is recognized in the interstitium adjacent to a central vein. **c**, Elastica van Gieson staining demonstrates a vessel (arrowheads) containing erythrocytes in the lumen and running alongside the interlobar vein. **d**, Figure showing the same area as (**c**) in a serial section. A vessel (arrowheads) containing erythrocytes found in (**c**) is positive for D2-40, which indicates that it is a lymphatic vessel. **e**, The arcuate vein in the centre of the figure shows positivity for CD31. Capillaries adjacent to the vein are also immunopositive for CD31 (arrows). **f**, Figure showing the same area as (**d**) in a serial section immunostained with D2-40. Note two capillaries (arrows) positive for D2-40, which are also positive for CD31, as shown in (**e**).

Lymphatics were abundant in the interstitium around the interlobar arteries and ran alongside them (Figure 3a). The interlobar veins were recognized in the vicinity of the interlobar arteries and lymphatics were distributed not only in the interstitium around the veins but also in their walls (Figure 3b). The latter were distributed in both the media and intima just under the endothelium. Large blood vessel-related lymphatics generally had empty lumina, but a few lymphocytes or erythrocytes were sometimes present within them (Figure 3c,d).

The arcuate arteries/veins were also accompanied by lymphatics, whose distribution was similar to that in the interlobar arteries/veins. Most of the large blood vessel-related lymphatics were immunopositive not only for D2-40 but also for CD31 (Figure 3e,f).

#### Distribution of lymphatics in relation to renal anatomical structures

**Figure 4:**
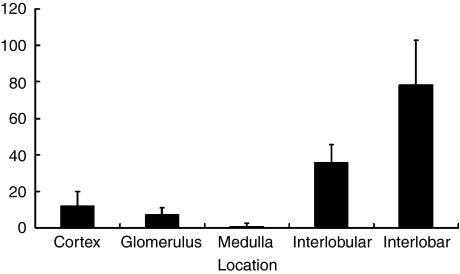
Lymphatic distribution in the normal kidney in relation to anatomical structures. This figure shows the number of lymphatics in 20 high-power fields (× 200) in 10 cases with normal structures. The values are 12.0 ± 7.9 in the interstitium between the tubules in the cortex (Cortex), 7.4 ± 3.6 in the interstitium around the glomeruli (Glomerulus), 0.8 ± 1.7 in the interstitium of the medulla (Medulla), 36.0 ± 9.4 in the interstitium around the interlobular arteries/veins (Interlobular) and 78.3 ± 24.5 in the interstitium around the interlobar arteries/veins (Interlobar). The lymphatics are abundant in the interstitium around the arteries/veins. Hardly any lymphatics are evident in the medulla. Statistical analyses (Student's *t*-test) between all location groups revealed a significant difference of *P* < 0.0001 except for comparison between ‘Cortex’ and ‘Interlobar’, which was *P* = 0.0236.

The extent of lymphatic distribution in relation to anatomical structures is shown in Figure 4. Lymphatics were distributed mainly in relation to the course of blood vessels and were observed sporadically in the parenchymal interstitium.

#### Renal pelvis

In the pelvic interstitium beneath the transitional epithelium, lymphatics were scattered in the same way as blood vessels. Lymphatics were also recognized in adipose tissues of the hilum and abundant in the interstitium around the renal hilar artery/vein.

### Lymphatics in end-stage kidney from haemodialysis patients

**Figure 5:**
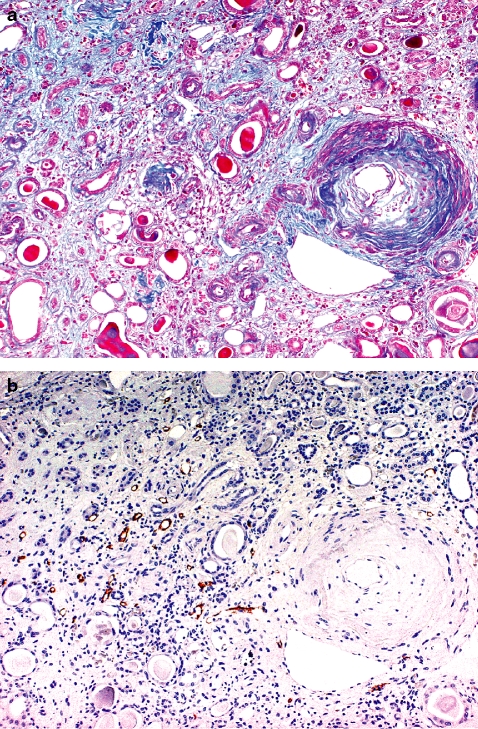
Lymphatics in end-stage kidney. **a**, Azan–Mallory staining of an end-stage kidney demonstrates severe atrophy of the cortex. The interlobular artery exhibits fibrous thickening of its wall. **b**, Figure showing the same area of a serial section of (**a**). In the interstitium of the cortex, lymphatic capillaries positive for D2-40 are abundantly distributed, but artery-related lymphatics are scarce.

**Figure 6:**
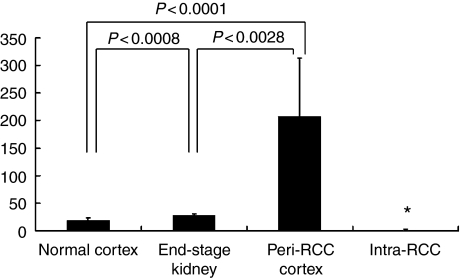
Comparison of lymphatic distribution in the normal cortex with that in end-stage kidney, fibrous cortex around renal cell carcinoma (RCC) and the intra-RCC area.The number of lymphatics in 20 high-power fields (× 200) is shown for four categorized locations. The values are 18.6 ± 4.6 in the normal cortex (*n* = 10), 27.6 ± 2.6 in the cortex of end-stage kidney (*n* = 3), 207.7 ± 105.9 in fibrous cortex around RCC (peri-RCC cortex; *n* = 10) and 0.8 ± 1.5 in the stroma of RCC (intra-RCC; *n* = 10). The number of lymphatics is significantly higher in the cortex of end-stage kidney than in the normal kidney. In RCC cases, the peri-RCC cortex shows abundant lymphatic distribution that is significantly more extensive than that of normal kidney, but lymphatic vessels are seldom detected in the area of intra-RCC. *The value for the intra-RCC area is significantly lower (*P* < 0.0001) than those for the other three locations.

End-stage kidney exhibited severe atrophy with a fibrous interstitium, sclerotic glomeruli and atrophic tubules. Interlobular arteries were closely distributed with severe fibrous thickening of their walls. Lymphatics were scattered in the cortical interstitium with a relatively higher density than that in the normal kidney (Figure 5a,b). In the fibrous interstitium around the interlobular arteries, lymphatics were more scarce than in the normal kidney. The medulla also possessed fibrous interstitium where lymphatic capillaries were never evident. The number of lymphatics in 20 fields of view (× 200) was significantly greater in the cortex than in normal cortex, as shown in Figure 6.

### Lymphatics in the kidney affected by rcc

**Figure 7:**
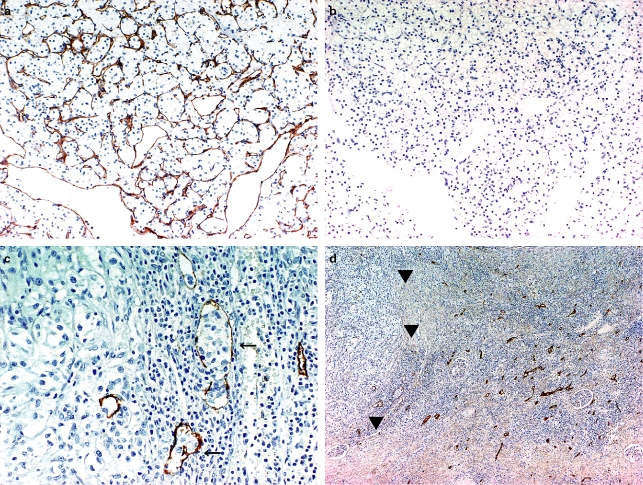
Immunohistochemistry of kidney with renal cell carcinoma (RCC). **a**, The central area of RCC has abundant blood vessels positive for CD31. **b**, Figure showing the same area in a serial section of (**a**). D2-40 immunostaining reveals a negative result. **c**, At the tumour margin, two lymphatics positive for D2-40 are invaded by tumour cells (arrows). **d**, In the fibrous interstitium around the tumour, lymphatics positive for D2-40 are abundantly distributed. Arrowheads indicate the boundary between the tumour and the tumour-free cortex.

Ten cases of RCC commonly formed a nodular mass with a fibrous capsule and microscopically exhibited clear cell carcinoma. In the tumour, blood capillaries positive for CD31 were abundant in a scanty stroma (Figure 7a). On the other hand, lymphatic vessels positive for D2-40 were not detected in the central area of the tumour (Figure 7b) and only a few lymphatics were evident in the tumour margin near the fibrous capsule. In two cases, a few lymphatics invaded by RCC were clearly detectable at the tumour margin or in the vicinity of the tumour (Figure 7c). The tumour-free cortex around RCC exhibited interstitial fibrosis with tubular atrophy, where lymphatics were abundant (Figure 7d), and some of them contained erythrocytes in their lumina. The cortex distant from the tumour showed the same distribution pattern of lymphatics as that seen in normal kidneys.

The number of lymphatic vessels in 20 high-power fields (× 200) was markedly greater in the fibrous cortex including the capsule than in the normal cortex (Figure 6). On the other hand, the number of lymphatics in the tumour was significantly lower than in the normal cortex.

### Lymphatics in kidneys with infarction, acute tubular necrosis and hydronephrosis

In a case showing fresh infarction, no lymphatic capillaries were observed in the lesion, but the distribution pattern of lymphatics around the lesion was similar to that in the normal cortex. Old infarcts with replacement fibrosis and many sclerotic glomeruli had few lymphatics. Lymphatics around small blood vessels in old renal infarcts were sparse. In a case with acute tubular necrosis, lymphatic capillaries were detected in the cortical interstitium and the lymphatic distribution around the interlobular arteries/veins was similar to that in the normal kidney.

The renal cortex in the kidney affected by hydronephrosis showed marked atrophy with a fibrous interstitium, sclerotic glomeruli and atrophic tubules. The medullary interstitium also exhibited fibrosis. Lymphatic vessels were abundant in the interstitium around the interlobar and arcuate blood vessels. However, the number of lymphatics around the interlobular arteries/veins was relatively lower than that in normal kidney. In the cortical fibrous interstitium, a few lymphatic capillaries were scattered, but lymphatic capillaries were absent in the medulla.

## Discussion

The distribution of lymphatics in the normal human kidney has not been well documented by light microscopy because of the lack of a reliable lymphatic endothelial-specific marker. The present study demonstrated that conventional immunohistochemistry using anti-D2-40 antibody is useful for the detection of renal lymphatics under both normal and pathological conditions and that the distribution patterns of lymphatics in relation to anatomical structures in the normal human kidney are similar to those previously reported in animals.^2^–^4^

In the present study, lymphatic vessels were abundant around the intrarenal arteries/veins and lymphatic capillaries were scarce in the interstitium around the glomeruli or between the tubules. Previous studies using animals have demonstrated that lymphatic capillaries are usually present in the vicinity of glomeruli and around the interlobular blood vessels^12^ and that large-calibre lymphatics are generally associated with the interlobular blood vessels.^2^–^4^ Microradiography of human kidneys has shown that lymphatics begin in the cortical interstitium near glomeruli and run adjacent to afferent arterioles and interlobular arteries.^5^ Our present results are in accord with those previous reports^2^–^5^,^12^ and suggest that the lymphatic system begins in the cortical interstitium around the glomeruli or between the tubules.

The existence of intramedullary lymphatics has been controversial. There is no lymphatic system in the renal medulla in dogs,^13^ but a few lymphatic capillaries have been observed in the medulla of pigs and humans.^4^,^6^ The present study revealed that a few lymphatic capillaries were also distinct in the margins of the medulla in four normal kidney specimens, although the number of lymphatics was markedly lower in the medulla than in the cortex. This difference with regard to the presence of medullary lymphatics among individual cases was probably due to the small number of tissue samples studied.

The present study revealed the sporadic presence of erythrocytes in the lumina of lymphatic vessels around the interlobar and arcuate arteries/veins or in the kidneys under pathological conditions. These vessels were immunopositive not only for D2-40 but also for CD31. CD31 is immunoreactive with the endothelium of both blood vessels and lymphatics^14^ and D2-40 consistently discriminates the lymphatic endothelium from the blood vessel endothelium.^8^ In addition, the lumina of human peripheral lymphatics sometimes contain erythrocytes, lymphocytes and macrophages, especially when there is peripheral congestion.^15^ In sheep, renal lymph also includes more erythrocytes than lymph collected from other organs.^16^ Thus, the lymph system plays an important role in the traffic of blood cells from the periphery to lymph nodes, which are a site of catabolism of senescent erythrocytes.^16^,^17^ Considering these facts, the existence of erythrocytes in lymphatics is not surprising and D2-40+ vessels with erythrocytes in their lumina should still be considered as true lymphatics. In haematoxylin and eosin-stained sections, pathologists often regard vessels with erythrocytes in their lumina as capillaries. Our present results appear to indicate that the distinct differentiation of lymphatics from blood capillaries in tissue sections requires adequate staining, such as D2-40 immunostaining.

In this study, D2-40 immunostaining was also helpful for detecting lymphatics in cases of RCC. A few lymphatic vessels invaded by RCC cells were sporadically detected at the tumour margin. Microvascular invasion by RCC cells is significantly associated with regional lymph node metastasis^18^ and is the most relevant prognostic feature after nephrectomy for RCC.^19^ Confirmation of vascular invasion in RCC may represent an essential histopathological feature for prediction of patient outcome. However, discrimination between lymphatics and blood capillaries has, thus far, not been investigated in RCC cases with microvascular invasion. Our present results indicate that immunohistochemistry for D2-40 makes it possible to investigate the relationship between lymphatic invasion by RCC cells and prognosis.

In the present study, the number of cortical lymphatics around RCC was markedly increased. Tumour cells or tumour-related macrophages generally produce lymphatic endothelial growth factors, such as vascular endothelial growth factors C and D.^20^ The abundant lymphatic distribution around the tumour revealed by this study may have been due to the effects of such endothelial growth factors. Our study also revealed abundant lymphatics in the cortex of end-stage kidney. This apparent increased density of lymphatics may not represent true proliferation of lymphatics but a deceptive superficial increase in lymphatic density, because end-stage kidney exhibits a compact distribution of blood vessels accompanied by abundant surrounding lymphatics. Studies using D2-40 immunoreactivity may provide an opportunity to investigate further a possible pathophysiological role of lymphatics in renal diseases.

## Abbreviation

RCC: renal cell carcinoma
